# Malaria Molecular Epidemiology: Lessons from the International Centers of Excellence for Malaria Research Network

**DOI:** 10.4269/ajtmh.15-0005

**Published:** 2015-09-02

**Authors:** Ananias A. Escalante, Marcelo U. Ferreira, Joseph M. Vinetz, Sarah K. Volkman, Liwang Cui, Dionicia Gamboa, Donald J. Krogstad, Alyssa E. Barry, Jane M. Carlton, Anna Maria van Eijk, Khageswar Pradhan, Ivo Mueller, Bryan Greenhouse, M. Andreina Pacheco, Andres F. Vallejo, Socrates Herrera, Ingrid Felger

**Affiliations:** Institute for Genomics and Evolutionary Medicine, Temple University, Philadelphia, Pennsylvania; Department of Parasitology, Institute of Biomedical Sciences, University of São Paulo, São Paulo, Brazil; Division of Infectious Diseases, Department of Medicine, University of California San Diego, La Jolla, California; Department of Immunology and Infectious Disease, Harvard T. H. Chan School of Public Health, Boston, Massachusetts; School of Nursing and Health Sciences, Simmons College, Boston, Massachusetts; The Broad Institute, Cambridge, Massachusetts; Department of Entomology, Pennsylvania State University, University Park, Pennsylvania; Laboratorio de Malaria, Laboratorios de Investigación y Desarrollo, Facultad de Ciencias y Filosofía, Universidad Peruana Cayetano Heredia, Lima, Peru; Institute de Medicina Tropical, Universidad Peruana Cayetano Heredia, Lima, Peru; Department of Tropical Medicine, Tulane University School of Public Health and Tropical Medicine, New Orleans, Louisiana; Division of Infection and Immunity, Walter and Eliza Hall Institute of Medical Research, Melbourne, Australia; Department of Medical Biology, University of Melbourne, Parkville, Australia; Department of Biology, Center for Genomics and Systems Biology, New York University, New York, New York; National Institute of Malaria Research, Odisha, India; Barcelona Centre for International Health Research, Barcelona, Spain; Department of Medicine, University of California San Francisco School of Medicine, San Francisco, California; Caucaseco Scientific Research Center, Cali, Colombia; Swiss Tropical and Public Health Institute, Basel, Switzerland; University of Basel, Basel, Switzerland

## Abstract

Molecular epidemiology leverages genetic information to study the risk factors that affect the frequency and distribution of malaria cases. This article describes molecular epidemiologic investigations currently being carried out by the International Centers of Excellence for Malaria Research (ICEMR) network in a variety of malaria-endemic settings. First, we discuss various novel approaches to understand malaria incidence and gametocytemia, focusing on *Plasmodium falciparum* and *Plasmodium vivax*. Second, we describe and compare different parasite genotyping methods commonly used in malaria epidemiology and population genetics. Finally, we discuss potential applications of molecular epidemiological tools and methods toward malaria control and elimination efforts.

## Introduction

Malaria-endemic areas have traditionally been classified in terms of transmission intensity, from hypo- to holoendemic. However, malaria epidemiology cannot be characterized solely on a one-dimensional scale. Prevalence of specific clinical manifestations of disease or parasite species, the onset of natural immunity, the spread of antimalarial drug resistance, and vectors involved in transmission, among others, vary across endemic areas.^1^,^2^ In the context of such complexity, the International Centers of Excellence for Malaria Research (ICEMR) network is taking advantage of state-of-the-art molecular tools to better characterize malaria epidemiology.

The ICEMR provide an opportunity to follow endemic areas over time and space, either longitudinally or by successive cross-sectional sampling at different spatial scales. These site-based research projects generate information and resources at regional scales, including epidemiologically contextualized molecular data and specimens, with the expectation that globally generalizable knowledge will emerge and guide evidence-based malaria elimination programs.

## The Malaria Epidemiologic Landscape: A Molecular View

Molecular methods have been used in malaria epidemiology for almost two decades.^3^ Although the initial emphasis was on diagnostics and genotyping, current epidemiological investigations have been enriched by incorporating population biology and population genetics modeling and concepts, which move the field beyond simple descriptions of malaria incidence and prevalence.

From an operational point of view, molecular tools allow 1) more sensitive estimations of prevalence and incidence that include subclinical cases of parasitemia; 2) assessment of the effectiveness of intervention strategies on the occurrence, complexity, and duration of infections; 3) differentiation between recrudescent, relapsing, and new infections; 4) estimation of the effect of interventions on the allele frequency of the targeted gene (e.g., mutations associated with drug resistance or variants in a vaccine construct); 5) estimation of the differential contribution of individual hosts to transmission by targeting gametocyte-specific genes; and 6) assessment of demographic patterns within parasite populations (gene flow–migration–colonization of new areas and population expansions), especially when transmission is driven by specific groups of particularly mobile subclinically infected individuals or migration across borders.^3^ This information, integrated with appropriate mathematical modeling or epidemiologic investigations, allows for the improvement of resource allocation and provide an early warning system to modify the intervention deployed in response to changing conditions.

A variety of coordinated efforts have focused on understanding geographic patterns of malaria transmission.^4^ Nonetheless, information derived from molecular data (e.g., malaria prevalence considering asymptomatic infections and/or parasite genetic diversity) still must be effectively integrated into standard reporting data to maximize public health benefit and facilitate assessment of interventions. Part of the problem resides in the challenge of integrating heterogeneous types of data at different temporal and spatial scales. Here we introduce efforts made across the ICEMR sites and discuss the challenges of incorporating these new technologies and concepts into evidence-based malaria control and elimination programs.

## Assessing Malaria Prevalence and Transmission

Diagnostics remain fundamental to molecular epidemiology. Although many specific aspects of malaria diagnostics are discussed separately in this supplement,^5^ here we address those issues that relate to disease ecology and transmission dynamics.

Light microscopy based on Giemsa-stained thick blood smears has limited the sensitivity for detecting low-density infections, hampering its use for measuring actual transmission.^5^ This situation will likely worsen wherever control efforts successfully reduce the incidence of clinical cases to the point where submicroscopic/subclinical parasitemia becomes relatively more common. For example, up to 73% of *Plasmodium vivax* infections were missed by microscopy, as compared with molecular detection, in hypoendemic areas of Brazil as the incidence of clinical cases declined.^6^ Similar reports are coming from all ICEMR projects where different diagnostic methods have been used.^5^ Rapid diagnostic tests (RDTs) are considered as an alternative to microscopy, but their utility is limited in some regions (e.g., Peru) because of the high frequency of parasites lacking *pfhrp2* and/or *pfhrp3* genes (the antigens targeted by the RDTs) in the Americas,^7^ and their low sensitivity in the face of low parasitemia, especially with *P. vivax*.^6^ To address this biological challenge, the ICEMR network has been testing a variety of nucleic acid amplification (NAA) methods in different epidemiological settings.^5^ NAA methods are costly when compared with microscopy, and some involve fairly complex laboratory resources that remain challenging in endemic areas. The ICEMR is developing strategies to circumvent this problem via field laboratories or mobile molecular laboratories (e.g., in non-Amazonian areas of the Americas or in Asia).^6^,^8^ Those experiences aim to facilitate the use of NAA methods in active case detection and reactive surveillance, but remain to be implemented as standard surveillance practice for most control programs.

In addition, the ICEMR is developing new approaches to detecting low-level malaria parasite infections. At the southwest Pacific ICEMR, two new quantitative polymerase chain reaction (qPCR) assays have been developed for *Plasmodium falciparum* that target repetitive genomic sequences, substantially increasing diagnostic sensitivity without requiring laborious sampling of large blood volumes or elaborate sample processing.^9^ Applying these ultrasensitive PCR assays in cross-sectional surveys in Tanzania and Papua New Guinea revealed a 10% higher prevalence rate compared with a standard qPCR assay.

## Gametocytemia

Determining the different contributions of groups of patients (e.g., clinical, asymptomatic) to malaria transmission requires information about gametocytemia. Asymptomatic infections, for example, represent a “hidden” parasite reservoir that can sustain transmission.^10^ Analysis of the effects of asymptomatic and submicroscopic infections on transmission must also consider differences among vectors, since vector transmission efficiency may vary with very low parasitemia. Furthermore, each malaria species poses a different set of challenges. ICEMR projects are investigating and comparing such differences across epidemiologic settings worldwide by using molecular tools.

The relationship between asymptomatic *P. falciparum* infections and parasite infectivity to mosquitoes has been investigated in Africa,^11^,^12^ but little is known in this regard about *P. vivax* or low endemic regions outside Africa, where other vector species are involved in malaria transmission. The two Latin American ICEMR sites have characterized *P. vivax* transmission dynamics in symptomatic and asymptomatic volunteers. Molecular assays (i.e., quantitative reverse transcription PCR [qRT-PCR]) found comparable expression levels of Pvs25 in symptomatic and asymptomatic volunteers.^13^,^14^ Blood from asymptomatic, low-parasitemia volunteer donors was able to infect laboratory-reared *Anopheles* mosquitoes (*Anopheles albimanus*) without statistically significant differences between direct feeding and membrane-feeding assays (S. Herrera, personal communication) indicating that asymptomatic carriers can infect mosquitoes in this setting. Studies to quantify the transmissibility of subpatent and asymptomatic parasitemia to *Anopheles darlingi* in Peru are ongoing (J. Vinetz and others, personal communication).

A major challenge to understanding parasite population biology and epidemiology is the paucity of data characterizing the different elements of intra-host dynamics.^15^–^17^ The southwest Pacific ICEMR has developed a novel multispecies/multistage approach that allows blood stage and gametocyte quantification of multiple *Plasmodium* species by qPCR and qRT-PCR.^18^ Furthermore, to understand intra-host dynamics (i.e., differential contribution of clones to gametocyte production, as well as possible within-host competition), a panel of highly polymorphic markers is being evaluated for genotyping *P. falciparum* gametocytes, including new and existing markers (e.g., *pfs230* and *pfg377*).^19^ Analogous highly polymorphic gametocyte markers for *P. vivax* have not yet been identified.

## Malaria Genotyping: Molecular Epidemiology and Population Genetics

Understanding the genetic diversity and structure of malaria parasite populations is the key for predicting the emergence and spread of phenotypes of interest, such as new antigenic or drug resistance variants. Whereas population genomics is an area of investigation reaching maturity in malaria,^20^,^21^ a wide variety of genotyping methods are still largely used to sample the parasite genome.^3^,^22^ Many of these methods have been used and compared across the ICEMRs sites worldwide.

Traditional genotyping methods for malarial parasites rely on the size polymorphism of genes encoding surface antigens with variable number of tandem repeats, such as *msp2* in *P. falciparum*, *msp3*α in *P. vivax*, and *msp1* and *csp* in both species.^3^,^23^–^26^ This approach has been useful to determine the number of different parasite genotypes coinfecting a single patient or multiplicity of infection (MOI) (see Table 1).^23^,^27^,^28^ However, it is of limited utility for interpreting other patterns (e.g., geographic population structure) since fragment sizes may converge at the population level, and the size itself may be under selection.^29^–^31^ The problem worsens if genotyping involves use of restriction enzymes, as demonstrated by one of the ICEMR sites. Specifically, as a result of multiple insertion–deletion mutations and recombination, alleles that differ at the sequence level may yield the same restriction fragment length polymorphism pattern.^31^

A second approach involves using partial or complete gene sequences from nuclear or organellar genomes. Such analyses have usually aimed to better understand the diversity of a targeted gene under consideration as a vaccine candidate, or because it harbors mutations linked to drug resistance.^3^,^25^,^32^,^33^ However, many studies have aimed to understand global patterns of diversity, including gene flow and/or population structure.^34^–^36^ A clear advantage to this latter approach is that such data are comparable across sites; however, this strategy is costly.

Finally, a major trend across ICEMR is the use of multi-locus genotyping that targets non-antigenic loci (Table 1). This approach allows different aspects of population structure to be studied, such as linkage disequilibrium and gene flow, as long as the sampled loci are not linked to a gene under selection.^22^,^37^–^40^ Two types of markers are widely used: microsatellites^37^,^38^,^40^–^43^ and single nucleotide polymorphisms (SNPs).^39^,^44^,^45^ Both approaches document similar epidemiological processes.^37^,^38^,^46^ However, microsatellites have a higher mutation rate than SNPs, which allows detection of recent events.^47^,^48^ A problem with microsatellites, however, is that they evolve according to complex evolutionary models, and not all microsatellites are equally suitable worldwide.^38^,^43^

Microsatellites are highly abundant in the *P. falciparum* genome; an average of one microsatellite locus is found per 2–3 kb of sequence. Microsatellite genotyping has revealed a wide range of population structures in *P. falciparum* isolates from four continents. Diversity and recombination rates are highest in holoendemic Africa,^49^,^50^ lowest in the hypoendemic areas of Central and South America,^38^,^49^,^51^ and intermediate in southeast Asia^49^,^52^ and Papua New Guinea.^53^ Although the observed recombination rate is related to transmission since it is affected by the inbreeding rate (see below under “Transmission intensity and molecular patterns”), diversity itself does not have a linear relationship with transmission, as it can be affected by (among other factors) historical processes.^3^,^54^ Despite the fact that only 160 microsatellites have been found in the genome of *P. vivax*,^39^ microsatellite-based studies have provided valuable information on the genetic diversity of this species. Genetic diversity of *P. vivax* was found to vary worldwide, with highest levels in south and southeast Asia^55^ and southwest Pacific,^42^,^56^ and lowest levels observed in South America^38^,^40^,^41^ and South Korea.^57^

ICEMR has been working on standardizing different sets of microsatellite loci to mitigate problems derived from these complex patterns of evolution. Particularly important are the efforts by Amazonia and southwest Pacific ICEMR sites to standardize *P. vivax* microsatellite loci for use across sites. ICEMR India and the non-Amazonia Latin America ICEMR have developed their own sets of microsatellite loci that complement those identified by others.

The high reproducibility of SNPs allows global comparisons and exploration of patterns over long time scales.^22^,^39^ However, ascertainment bias is a problem in some contexts; SNPs may be identified in a relatively small sample size, and may be more likely to reflect common rather than rare alleles,^58^,^59^ affecting assumptions in some population genetic analyses (e.g., inferences about parasite demographic history or selection). Thus, investigators should account for this bias when analyzing their data in such contexts.

As with microsatellites, multiple ICEMR sites (southwest Pacific, India, Amazonia, and Africa) are developing SNP typing protocols for *P. falciparum* and *P. vivax* to identify SNPs that can distinguish between parasites from different geographic areas. These “region-specific SNPs” will provide a means to predict the origins of outbreaks and to estimate the contribution of imported infections to overall transmission in areas where transmission has decreased to very low levels. The southwest Pacific ICEMR is developing algorithms to identify the most informative markers so that minimal numbers of markers can be developed as a parasite “barcoding tool”; other ICEMR sites are expected to follow suit. Regional molecular barcodes also include elements of the global barcode^44^ so that comparisons with regions outside the Pacific can be made.

The previously established term “molecular barcode” refers to a small standardized set of SNPs used for genotyping.^44^ An SNP barcode could permit tracking multi-locus genotypes in time and space provided the local transmission dynamics or history yield stable SNPs.^39^,^44^,^46^ On the other hand, recombination between different parasite clones will break such multi-locus genotypes. Many of the SNPs currently used in *P. falciparum* have been selected from different chromosomes so that demographic processes including origin of an infection can be studied.^44^ Similar to other approaches, however, the use of a SNP barcoding tool is challenged by a high proportion of multi-clone infections^3^ and problems related to ascertainment bias.^58^,^59^

## How are Genotyping Methods Applied?

### Individualization: does sample X match sample Y?

In clinical trials of antimalarial drugs, genotyping can help to distinguish between parasite recrudescence (i.e., the original parasites remain detectable despite antimalarial treatment) and new infections (i.e., blood-stage parasites detected after antimalarial treatment are genetically distinct from those present before treatment). A problem reported by ICEMR in hypoendemic areas was that the circulating parasites may be closely related, making it difficult to separate recrudescences from new infections.^38^,^46^,^60^ Furthermore, where the complexity of infection (i.e., number of different clones being transmitted) is high, relapse from hypnozoites (in the case of *P. vivax*) or recrudescence of a previously undetected minority clone may lead to a false conclusion.

### Monitoring the infection dynamics of parasite clones over time.

A novel application developed by the southwest Pacific ICEMR follows individual *P. falciparum* or *P. vivax* genotypes over time to determine the number of new infections on a background of preexisting parasite clones. This molecular measure of the force of infection (defined as the number of distinct parasite clones acquired over time) provides a marker for individual exposure and transmission and thus is suitable for measuring outcomes of interventions.^27^,^28^

### Relatedness: are these individuals related?

Determining whether all infections had a single origin (outbreak) or multiple origins is crucial for designing proper containment strategies, and DNA analysis can help in this task.^22^ Furthermore, it is essential in the case of urban malaria or reactive case detection where separating local from imported cases can dramatically change the interpretation of the results. This interest in tracking parasites in space and time is shared by a number of the ICEMR sites. As an example, short-term spread of a single clone or a few clones originating from a malaria outbreak was tracked in an area of declining malaria transmission in rural Amazonia.^40^ Other studies in Central and South America have also shown clonal or epidemic expansions of malaria parasite populations.^38^,^46^

A common theme across the ICEMR settings is that the number and type of loci needed for genotyping should be tailored to the objective of the epidemiologic investigation in the setting where such studies will be carried out. For example, a global study of *P. vivax* microsatellites carried out by the southwest Pacific and Amazonia ICEMR sites included 841 isolates from four continents collected in 1999–2008, which were genotyped with 11 microsatellite markers.^61^ In the context of their investigation, three loci were sufficient to identify 90% of all haplotypes. However, studies conducted by the non-Amazonia Latin America ICEMR in areas with hypoendemic malaria have shown that multi-locus-linked genotypes in both *P. falciparum* and *P. vivax* can be maintained over time, requiring a higher number of hypervariable microsatellites to achieve the same discrimination.^38^ This observation suggests that in hypoendemic malaria areas, numerous malaria cases may be infected by highly related parasites.^38^,^46^,^60^

### Gene flow and population structure.

Population structure is the result of common processes in nature, including inbreeding and geographic isolation.^3^,^32^,^33^,^35^,^36^,^38^,^54^,^56^ Such genetic structures are detectable at time scales determined by the locus-type mutation rates.^38^ Many ICEMR sites are standardizing different approaches to study population structure using both SNPs and microsatellite loci. An important trend in the ICEMR sites outside Africa is the comparison of population structures in *P. vivax* and *P. falciparum* in the same endemic settings. Whereas in some areas the two parasites show similar patterns,^38^ in others there are clear differences.^41^,^42^ These differences are expected because population structures will be affected by the local evolutionary history of each parasite species.^35^,^54^

A collaborative study of the geographic population structure of *P. vivax* performed by the southwest Pacific and Amazonia ICEMR sites showed that parasite populations from southeast Asia, where transmission was intermediate but the migration of infected hosts was high, were more diverse than populations sampled from South America.^61^ The interpretation of these data reflected the fact that malaria was nearly eliminated in South America in the 1960s. On the other hand, studies carried out by the ICEMR sites from Asia and non-Amazonia Latin America using complete mitochondrial genome sequences have shown that the genetic diversity of *P. vivax* in the Americas, as a region, may be comparable to that in Asia and Oceania.^34^,^35^

Unlike Asia where human migration increases local genetic diversity, the combined effects of the geographic structure and the low incidence of *P. vivax* malaria in the Americas have resulted in patterns of low local but high regional genetic diversity where several populations are isolated from each other. Thus, if only a handful of populations were sampled in the Americas, one could observe low regional genetic diversity. However, when aggregated, *P. vivax* in South America is the result of a complex demographic history with limited gene flow within and among some regions.^32^,^35^ This pattern offers interesting perspectives in the context of malaria elimination in the Americas. If smaller geographic areas that are relatively isolated can be defined, these can be targeted by malaria programs as “elimination units” with limited risk of reintroduction. Turning these observations into operationally relevant information will be a matter of defining the spatial connectivity and the factors leading to the observed gene flow at a time scale usable for elimination. Also, low gene flow between areas should facilitate containment in the event of the emergence of drug resistance. The exploration of these patterns will be accelerated by population genomics.^20^,^21^

### Transmission intensity and molecular patterns.

When interventions lead to reduction in transmission, it is expected that parasite population diversity, overall, will be reduced. This process will yield signatures that can be captured with molecular data (Table 2). There are two major approaches: 1) monitoring the reduction in MOI or the number of multiclonal infections and 2) monitoring changes in genetic diversity and in the parasite population structure (Table 2). These indicators show how transmission affects the parasite genetic diversity in a given population. However, they do not measure exactly the same processes.

MOI is the result of two ecologically distinct processes that are hard to differentiate by genotyping only: coinfections (two or more genotypes being transmitted simultaneously by a mosquito) and superinfections (a patient acquiring multiple but independent infections). Both of these processes relate to transmission intensity.^62^ Overall, the number of coinfections/superinfections is expected to positively correlate with transmission.^3^,^49^,^62^

Consistently, cross-sectional and longitudinal studies of *P. falciparum* indicate that the prevalence of multiclonal infections diminishes with a reduction in malaria transmission.^3^,^49^,^50^,^62^,^63^ This pattern seems to hold in all ICEMR sites for *P. falciparum*, but an analysis across sites is still pending. Despite this clear trend, the relationship between MOI and transmission is not linear,^3^ as transmission is not homogeneous, but occurs in hot spots depending on microscale differences in mosquito biting rates.^64^ Furthermore, a similar trend of reduction in MOI in low-transmission settings has not been observed for *P. vivax*, where multi-clone infections remain common even in low-transmission areas (Table 1).^37^,^38^,^55^,^56^ This could be the result of hypnozoites from prior infections accumulating in the liver and thus causing multiple relapses of distinct genotypes.^37^,^60^ More field studies are needed to understand the relatively high MOI in *P. vivax*, both in areas of intense transmission^37^,^56^ and in those approaching elimination.^38^,^55^ It is worth noting that the genotyping method used may affect estimates of MOI (see Table 1), so some standardization in methodologies and study design (e.g., age range) is required to compare results across sites/studies.

The second approach, evaluating changes in the parasite population structure and reduction in the parasite genetic diversity, focuses on looking for patterns consistent with an increase in the effect of genetic drift in the parasite population.^38^,^39^,^63^ This will lead to changes in allele frequencies and the expectation that genetic variation will be lost in response to declining transmission. More between-population divergence is expected to occur, leading to fragmented population structures. It is important to realize that the relationship between genetic variation and malaria transmission intensity is not linear simply because many patients could be infected by either related (e.g., as a result of inbreeding) or distinct parasites (see below in this section and Table 2).

As stated earlier, the proportion of infections comprised of a single genotype (monoclonal) is expected to rise when transmission decreases, so inbreeding is likely to increase.^3^ In areas with primarily single-clone infections (usually areas with unstable or low transmission), multi-locus genotypes are expected to persist in time and space. Thus, the number of infections by identical non-segregating genotypes and, as a consequence, linkage disequilibrium, is expected to increase.^38^,^39^,^49^,^63^,^65^ Indeed, it has been observed that wherever a dramatic increase in transmission occurred after a sustained decrease in malaria incidence, many infections are caused by identical or highly related parasites (a so-called clonal expansion).^3^,^38^ This approach (estimating linkage disequilibrium and frequency of infections with identical non-segregating genotypes), however, does not take into account rates and mechanisms of spontaneous mutations during an infection, which remain understudied and a high priority area of investigation. Furthermore, these investigations are difficult to perform in areas where multi-clone infections prevail.^3^,^37^,^49^,^62^

Estimates of genetic diversity may show even more complex patterns since they depend on the effective population size (see below in this section) and the mutation rate of the loci under study.^55^,^66^ One scenario is that, as a result of a reduction in transmission, many malaria cases could be caused by related (inbred) parasite lineages. However, changes in genetic diversity could be almost undetectable if there are a few genetically divergent inbred lineages coexisting in an area. These types of dynamics could explain, in part, the observed high genetic diversity across a broad transmission spectrum in both *P. falciparum* and *P. vivax* observed throughout the ICEMR sites and elsewhere.^38^,^39^,^55^,^66^ Indeed, heterozygosity seems to be less affected by population bottlenecks of short duration than by the number of alleles at a given locus.^67^ Evaluating the number of alleles is imprecise unless sample sizes are large, so it has not been widely used by the ICEMR sites.

Finally, some studies have explored estimating changes in the parasite effective population size, *N*_e_.^44^,^55^,^63^,^68^ This more abstract concept requires some discussion. *N*_e_ is not equivalent to heterozygosity but rather predicts loss in heterozygosity. It relates to the uneven reproductive success of parasite lineages.^68^ Importantly, *N*_e_ has the properties of the harmonic mean, so its value is affected by the smallest population size.^68^ The *N*_e_ concept has important implications in malaria epidemiology if we consider that changes in genetic diversity in a set of loci are observable at a time scale that is relative to their mutation rates. For example, after a reduction in *N*_e_ that might occur after a sustained intervention, heterozygosity would be expected to recover faster at microsatellite loci than SNPs simply because the former have a higher mutation rate. Such a difference can be informative in terms of evaluating the long-term effect of interventions.

Although a reduction in the effective population size is expected if transmission is reduced, many factors could make such an outcome difficult to observe. First, there are different ways to measure *N*_e_, each one measuring different aspects (e.g., number of parents or differences in the number of progeny).^68^ Second, the relationship between *N*_e_ and malaria incidence is likely not linear as has been shown in other pathogens,^69^ for example, a high malaria incidence in a community with closely related parasites and a high variance in the number of new cases transmitted from infected individuals could still yield a parasite population with low *N*_e_. Third, *N*_e_ estimates might be inflated by migration or population substructure.^63^,^68^ Finally, since *N*_e_ has the property of the harmonic mean,^68^ the number of infections that sustains the parasite population between transmission seasons may have a greater impact on *N*_e_ than the total number of cases in a given year or the number of cases during the high-transmission season.^63^ Thus, epidemiologically relevant changes in transmission may not be detectable in terms of a reduction in *N*_e_.

## Conclusions

The use of technologies that capture genetic information from the parasite, vector, and patients permits characterization of malaria transmission in ways that were unthinkable 10 years ago. That intra-host dynamics characterized by a combination of RNA- and DNA-based measurements illustrates major advances in malaria epidemiology from incorporating molecular tools. The ICEMR sites are currently taking advantage of such methods to better characterize malaria transmission across study sites with divergent epidemiologic characteristics. How to translate complex population genetic data into epidemiologically relevant information on malaria transmission is an ongoing discussion in the ICEMR network. Molecular methods provide information on malaria prevalence/incidence that considers subclinical/asymptomatic infections and better characterizes gametocytemia. Population genetic parameters will likely provide useful information if they are interpreted properly; nevertheless, population genetic approaches require further validation by evidence gathered by all ICEMR sites and comparisons worldwide.

## Figures and Tables

**Table 1:** Genotyping methods used and prevalence of single-clone infections

ICEMR	Genotyping method	% Single-clone infections
Southeast Asia	*Pfmsp1*, *Pfmsp2*, *Pfglurp*	China–Myanmar border: *Pf* 54.3%*
	*Pf* microsatellites	Not yet determined
India	Next-generation sequencing	Not yet determined*
Southwest Pacific	*Pv* microsatellites, *Pvmsp1*, *Pfmsp1*, *Pfmsp2*, *Pfs230*, and *Pfg377* using PCR fragment sizing by capillary electrophoresis	Wosera, PNG: *Pv* 25% in *N* = 1,194; *Pf* 66% in *N* = 1,868
West and central Africa	Barcode based on 24 SNPs	Thiès, Senegal: *Pf* > 90%
		Gambissara, The Gambia: *Pf* 30–40%*
		Dangassa, Mali: *Pf* 15–20%*
Amazonia	*Pf* and *Pv* microsatellites	Remansinho, Brazil: *Pv* 21.5%
Non-Amazonia Latin America	*Pf* and *Pv* microsatellites	Tierralta, Colombia: *Pf* 67.6% in *N* = 34; *Pv* 60.3% in *N* = 262
		Tumaco, Colombia: *Pf* 81.1% in *N* = 148; *Pv* 34.3% in *N* = 67

Parasite species: *Pf* (*P. falciparum*), *Pv* (*P. vivax*); Loci: *Pfmsp1* = *Pf* merozoite surface protein 1, fragment size polymorphism in the block 2 region within allele families; *Pfmsp2* = *Pf* merozoite surface protein 2, fragment size polymorphism within allele families; *Pfglurp* = *Pf* glutamate-rich protein, fragment size polymorphism; *Pvmsp1* = *Pv* merozoite surface protein 1, fragment size polymorphism; *Pfs230* = *Pf* gametocyte specifically expressed gene, fragment size polymorphism; *Pfg377* = *Pf* gene expressed in female gametocytes only, fragment size polymorphism; PCR = polymerase chain reaction; PNG = Papua New Guinea; SNPs = single nucleotide polymorphisms.

* Work in progress.

**Table 2:** Molecular criteria used to measure the expected parasite population decline as a result of a reduction in malaria transmission

Metric	Expectation	Limitations
Prevalence of multi-clone infections	Decreases with declining transmission	Sensitive to the resolution of genotyping method and loci used
Linkage disequilibrium	Reflects an increasing inbreeding rate, and increases with declining transmission	Cannot be measured in multi-clone infections and depends on variation in genotyped loci
Prevalence of infections with identical or related nonrecombinant genotypes	Reflects an increasing inbreeding rate, and increases with declining transmission	Not suitable in settings with high prevalence of multi-clone infections
Number of alleles (genetic diversity)	Reflects reduction in effective population size, and decreases with declining transmission	Sensitive to sampling bias (e.g., low-frequency alleles require unrealistic sampling efforts)
Heterozygosity (genetic diversity)	Reflects reduction in effective population size, and decreases with declining transmission	Requires a sustained decrease in transmission to decline
Effective population size (*N*_e_)	Decreases with declining transmission	Sensitive to demographic processes (e.g., migration) and to the method used to estimate it. Declines after a sustained reduction in the parasite population below the minimum naturally occurring population size
